# Deep Feature Extraction and Classification of Android Malware Images

**DOI:** 10.3390/s20247013

**Published:** 2020-12-08

**Authors:** Jaiteg Singh, Deepak Thakur, Farman Ali, Tanya Gera, Kyung Sup Kwak

**Affiliations:** 1 Chitkara University Institute of Engineering and Technology, Chitkara University, Punjab 140401, India; jaiteg.singh@chitkara.edu.in (J.S.); tanya.gera@chitkara.edu.in (T.G.); 2 Department of Software, Sejong University, Seoul 05006, Korea; farmankanju@sejong.ac.kr; 3 Department of Information and Communication Engineering, Inha University, Incheon 22212, Korea

**Keywords:** convolutional neural network, malware, classification, android, security, visualization

## Abstract

The Android operating system has gained popularity and evolved rapidly since the previous decade. Traditional approaches such as static and dynamic malware identification techniques require a lot of human intervention and resources to design the malware classification model. The real challenge lies with the fact that inspecting all files of the application structure leads to high processing time, more storage, and manual effort. To solve these problems, optimization algorithms and deep learning has been recently tested for mitigating malware attacks. This manuscript proposes **S**umming of neur**A**l a**R**chitecture and **V**isualizati**O**n **T**echnology for **A**ndroid **M**alware identification (SARVOTAM). The system converts the malware non-intuitive features into fingerprint images to extract the quality information. A fine-tuned Convolutional Neural Network (CNN) is used to automatically extract rich features from visualized malware thus eliminating the feature engineering and domain expert cost. The experiments were done using the DREBIN dataset. A total of fifteen different combinations of the Android malware image sections were used to identify and classify Android malware. The softmax layer of CNN was substituted with machine learning algorithms like K-Nearest Neighbor (KNN), Support Vector Machine (SVM), and Random Forest (RF) to analyze the grayscale malware images. It is observed that CNN-SVM model outperformed original CNN as well as CNN-KNN, and CNN-RF. The classification results showed that our method is able to achieve an accuracy of 92.59% using Android certificates and manifest malware images. This paper reveals the lightweight solution and much precise option for malware identification.

## 1. Introduction

Any software with mala fide intention is a malware (malicious software). They generally have a mischievous behaviour and are developed to interrupt normal functioning, steal sensitive information, display unwanted advertising, or getting control of the users’ device without knowledge. Moreover, malware and unintentionally harmful software are collectively termed as badware. Main categories in which malware can be grouped are the virus, worms, Trojans, ransomware, rootkits, and botnet [1]. Like computer systems, malware systems have evolved to be more intelligent, smart, and decisive. Malware can adopt polymorphic and metamorphic techniques to obfuscate traditional methods of malware identification  [2,3,4,5]. Newly developed malware is too sophisticated to obstruct emulators and avoid deep static analysis. Malware also propagates through deploying metamorphism methods like multi-packer, code transformation, encryption, registry modification, virtual machines, anti-debugging, and instruction permutation. Malware is smart enough to detect the best moment to launch its payload [4,6,7,8,9]. The use of automation and reusable development modules can generate a huge amount of new malware variants [10,11,12]

Malware developers tend to change small parts of the original source code to generate new malware variants and evade detection [11,13,14]. This makes identification of malware variants from the same family extremely challenging [15,16].

The most prominent signature and behavior-based techniques for malware identification are static and dynamic analysis. In static analysis, the suspected code is analyzed without running the application. It requires disassembly of source code for feature extraction [17,18,19,20,21,22]. It is not resilient against code obfuscation and dynamic code loading [23,24,25,26]. On the other hand, dynamic analysis examines the features and traces of suspected application while it is executing [27,28,29,30,31,32]. The latter technique is promising but complex and time-consuming. It is high on resource consumption and storage space [23,33]. Intelligent malware is using anti-emulation techniques to evade dynamic analysis [34,35,36]. Moreover, utilizing static and dynamic techniques on such files requires a lot of manual effort/human intervention. It also requires domain-level knowledge to analyze or reverse engineer the application [37,38,39,40,41]. The time required to manually construct the features from the whole Android Application Package (APK) structure for the classification of Android malware families is considerably high [4,6,42,43,44]. These security mechanisms demand high computational resources and deploying them on a constrained smartphone environment is difficult [34]. Android malware traces are studied through Classes.dex, resources, manifest, and certificate files of Android application. The real challenge lies with the fact that inspecting all files leads to high processing time, more storage, and manual effort. Optimization algorithms and deep learning has been recently tested for mitigating malware attacks.

A model can easily be trained using deep learning algorithms for malware identification. If supported with Graphics Processing Unit (GPU) acceleration such models can perform reasonably well to identify malicious behavior of any application. Such deep learning models with GPU support have proven to guarantee excellent performance for image classification. Deep learning network [45,46] such as Convolutional neural network (CNN) takes the images as input. It has the ability to differentiate various aspects/objects from one other by using learnable weights and biases. There is no better choice than CNN when the input data is in the form of images. CNNs eliminate the tasks such as feature engineering, features selection, and features representation that may require extensive human intervention. CNN has achieved promising results in real world research applications such as sentiment analysis, bioengineering, pedestrian detection, face recognition, and handwritten digit recognition. In the proposed work too, Android malware applications have been converted into malware images. Owing to the proven and widely accepted method for image classification by research community, a CNN was thus fine-tuned to automatically extract the rich features from malware images. These features were thus used to perform the classification of malicious applications with respect to their families. This methodology suggests the conversion of binary information from Android files into images. Such visualization-based techniques allow analysts to see through the malware binary images without executing it. Unlike machine learning, deep learning algorithms can perform feature representation without any specific assumption or parameter configuration. With little guidance, deep learning models can capture the right features, learn complex patterns, and effectively solve the dimensionality problem. The main contributions of this work are enumerated as follows:

- We propose a novel system called SARVOTAM that is defined as **S**umming of neur**A**l a**R**chitecture and **V**isualizati**O**n **T**echnology for **A**ndroid **M**alware classification.
- It works on the raw bytes and eliminates the need for decryption, disassembly, reverse engineering, and execution of code for malware identification. The system converts the malware non-intuitive features into fingerprint images to extract the quality information.
- Seeing through malware binary, the proposed system can discover and extract insights necessary for malware analysis, and paves the path for the development of effective malware classification systems.
- A CNN was fine-tuned to automatically extract the rich features from visualized malware thus eliminating the feature engineering and domain expert cost.
- SARVOTAM was augmented by imbuing traditional classifiers like K-Nearest Neighbour (KNN), Support Vector Machine (SVM) and Random Forest (RF) to recommend prominent Android File structure features for malware identification and classification. It was noted that CNN-SVM model outperformed original CNN as well as CNN-KNN, and CNN-RF.
- To the best of our knowledge, classification and generation of malware images using fifteen unique combinations of Android malware file structure have been explored for the first time.
- It was observed that malware images formed using Certificate and Android Manifest files (CR+AM) offer a light-weight and much precise option for malware identification. One may not try inspecting all files in the APK for malware identification and classification.
- The proposed system was evaluated against the DREBIN dataset [47]. This dataset consists of 179 different malware families containing 5560 applications.

**Figure 1:**
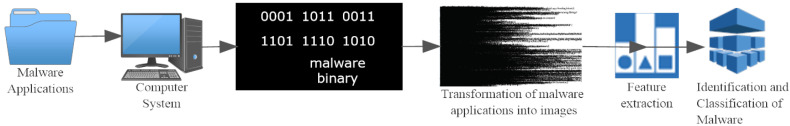
Simplistic depiction of adopted methodology for classification of Android malware.

The simplistic depiction of proposed SARVOTAM methodology is shown in Figure 1.

- Malware applications: Malicious applications from the DREBIN dataset were considered to evaluate the efficiency of the proposed methodology. This dataset contains 179 Android malware families and is widely used among the research community.
- Computer System: The machine with configuration Intel core i5 processor, 8G RAM, and 2.7 Ghz clock speed was used for the experiments and results.
- Transformation of malware applications into images: The proposed SARVOTAM system allows seeing through malware binary, discover and extract insights necessary for malware analysis by converting malware binary into grayscale images. Fifteen unique malware images were created using different files of an APK for every malware family samples. Section 3.1 discusses in detail about the methodology adopted to transform malware applications into images.
- Feature Extraction: Accurate Feature engineering is the important task for any classification model. In this study, a fine-tuned CNN was used to automatically extract rich features from visualized malware images thus eliminating the feature engineering and domain expert cost. Section 3.2.1 discusses more about CNN architectures, used in the experiments.
- Identification and classification of malware: The machine learning algorithms such as SVM, KNN, and RF were used for the classification purpose. More detail about this is presented in Section 3.2.2 and Section 4.

The rest of this paper is organized as follows; Section 2 offers a discussion on related work; Section 3 elaborates adopted methodology; Section 4 interprets the experimental results and Section 5 concludes the findings.

## 2. Related Work

Visualization-based analysis of malware has been conducted by the researchers [10,48,49,50]. Visualization-based approaches tend to directly work on malware image structure [11,51,52,53]. Unlike static and dynamic techniques, visualization-based analysis supports the faster classification of the malware samples as it does not require an application to be disassembled or executed. Therefore, it outperformed than conventional techniques when the task is to classify a large number of malware samples. In  [54], the author converted an APK file structure into four different image formats. Those image formats were Grayscale, Red-Green-Blue (RGB), Cyan-Magenta-Yellow-Black (CMYK), and Hue Saturation Lightness (HSL). Three different machine learning classifiers namely, Decision Trees, Random Forest, and K-Nearest Neighbour were trained using Global Image Descriptors (GIST) features against each image representation to classify whether an application is benign or malware. The authors achieved a high accuracy of 91% with random forest classifier on grayscale image representation. Authors in [11] performed fine-grained classification on Portable Executable (PE) files using the visualization-based approach. They visualize the malware as an RGB-coloured image. The dataset was composed of 15 families that contained 7087 malware samples. They built their model by combining global and local features for the malware classification. The data and code section of the file was processed as feature vectors to constitute local features. Global Features were extracted from RGB-coloured image. To train the model they used three classifiers namely, Random Forest, Support Vector Machine, and K-Nearest Neighbour. The results of the malware classification experiments showed that the Random Forest classifier achieved a high accuracy of 97.47%. Their approach did not work with a non-PE file structure, e.g., an APK file structure. Hence, their method cannot be used directly for classification Android malware families. Authors in [55], consider only the code section of an APK file. For this task, they first converted the dex file into a jar file using dex2jar tool. Further jar file was converted into java file using jad tool. For each APK file, they put the code part in separate text files. To identify the important words in text file, authors employed the technique called as Term Frequency-Inverse Document Frequency (TF-IDF) in their work. TF-IDF weight is a statistical measure that helps to interpret that how important a term is to a text file in a collection of large text files. TF computes the normalized term frequency, which is calculated as the number of times a term appears in a document, divided by the total number of terms in that document. IDF measures how important a term is. It is computed as the logarithm of the number of documents in the corpus divided by the number of documents where the specific term appears. It helps to weight down the frequent terms while scaling up the rare ones. After mining the important terms from the text files, they arranged these files into several groups. These groups were further processed to generate pictures by using simhash [56] and djb2 algorithm [57]. The authors deployed a convolutional neural network for learning and classification and achieved an accuracy of 92%. Authors ignored other building blocks of APK file, such as META-INF, Resources, AndroidManifest.XML in their work. Authors in [58], demonstrated the experiment over 32 malware families constituting 12,000 images of malware. They studied the performance comparisons on various classifiers such as a Convolutional Neural Network, K-Nearest Neighbour, and Support Vector Machine with different image descriptors such as Local Binary Pattern (LBP) and GIST. Convolutional neural network model trained with 6 layers using LBP features achieved a high accuracy of 93.92% against the dataset chosen. They visualized the malware as grayscale and Red Green Blue Alpha (RGBA) images. Researchers analysed the performance of both image formats using the CNN model, which was trained with LBP features. Authors also concluded that visualizing malware as a colour image might lose some important features. In machine learning, deciding the subset of features that can potentially be used for critical malware analysis is a challenging task. A proper feature set should be generated to build an accurate malware analysis or detection model. Authors in [59] developed the visualization method in C language to study the internal structure (patterns/anomalies) of Android malware executable files. Researchers also claimed that their method has the potential to disclose feature set for classification of malware families. They only considered the .dex file in their work. Bytes in .dex file were mapped to a pixel on the image. Numerous varieties of obfuscation tools have been available in the market, being used by legitimate developers to protect their intellectual property of Android applications. The tools and techniques which were originally designed to protect intellectual property are now widely exploited and abused among malware authors to create Android malware variants more resilient. Authors in [60] utilize the visualization-based approach to fingerprint the obfuscation tools used in the development of the Android application cycle. Malware binary visualized as an image. They calculated two types of statistical features from an image. These features are synthesized to extract information to uncover the type of obfuscation tool employed by an application developer. Researchers claimed accuracy of 73% and 86% for fingerprinting the obfuscation tool and classification of obfuscated and original applications respectively.

The literature review concludes the fact that an APK file is a sequence of bits and therefore a binary image, there is no clear consensus within researchers pertaining to type of analysis and prominent APK parameters suitable for the classification of malware entities. The traditional malware classification approaches rely on extracting static and dynamic features. These approaches tend to use code analysis to solve a malware classification problem. Existing malware classification approaches used signature-based and feature-based approaches. Unfortunately, these approaches suffer from code disassembly, code obfuscation, and high consumption of resources. Researches have also realized that these approaches are heavy on time and space. Moving towards deep learning infusion with visualization approaches is the beginning of a new era in Android security. The proposed solution leverages the goodness of visualization and deep learning techniques to solve the multiclass malware classification problem. Deep learning architecture eliminates the need to capture features such as API calls, permissions, meta-data information, and other dynamic features such as system call, network activity to generate a high-quality malware classification model. The solutions leveraging the combination of visualization-based analysis and deep learning [61,62] have shown the impact lately in the research related to security and privacy. Most of the proposed solutions [10,11,16] have attained good accuracy against windows malware classification. Researchers worked with PE files because their experiments were restricted to Windows environments. Windows platform is most popular in desktop personal computers, and their hardware architecture is much different from light-weight mobile devices running Android. Therefore, solutions for Windows platform applications such as PE files cannot be directly applied for Android malware family classification. The cited literature has been published in the year 2020 and the authors have probably not tested it on APKs. This study validates the use of feature extraction for Android malware images.

## 3. Materials and Methods

**Figure 2:**
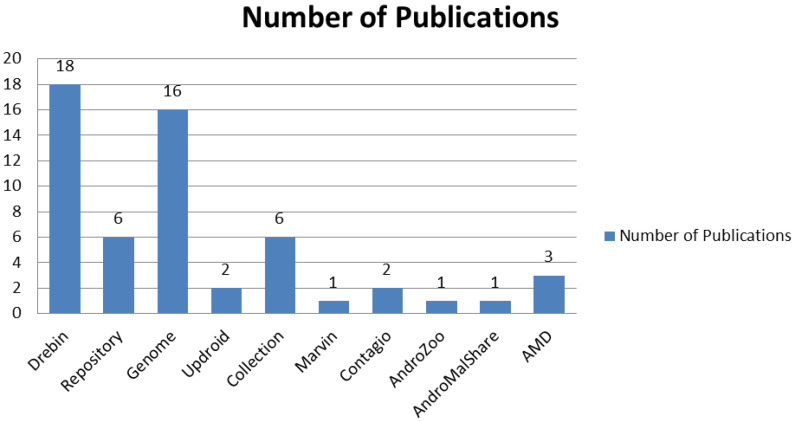
Popular Android malware datasets.

This section offers a discussion on various fundamental concepts involved in the experiment design. DREBIN dataset of Android malware applications has been used for this experiment. The dataset contains 5560 files from 179 different malware families. Most of the research literature from year 2014–2020 has used DREBIN dataset as standard dataset for malware related experiments. The dataset includes popular Android malware families such as Fake Installer, GoldDream [24], GingerMaster [23] and DroidKungFu [25]. A summary of malware datasets used by the research community is summarized in Figure 2 [63]. Further, the prime objective of this manuscript lies with validating the proposed method for malware identification instead of malware itself. Furthermore, the most recent malware dataset available for research is from year 2017 [12], which too may not have sufficient samples of contemporary malware types.

Experiment design, adopted methodology and fundamental contributory concepts are detailed next.

### 3.1. Transforming Malware APK into Images

**Figure 3:**
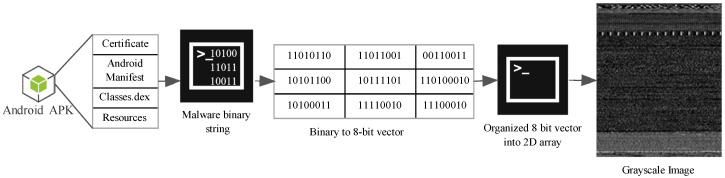
Conversion process of APK into grayscale image.

As per established research standards classes.dex, resource, manifest, and certificate files are primarily considered for visualization of APK [55]. In this manuscript, the authors generated malware images using these four files from malware APK. The malware binaries are converted into 8-bit vectors and subsequently converted into grayscale images. There are a few fundamental steps involved in transforming any malware samples into a digital image. Entire malware substring can be seen as the sequence of several substrings. Each substring is 8-bit length long and termed as a pixel. Further, this 8-bit substring is mapped to an unsigned decimal number within a range from 0 to 255. For example, if a bit string is 0011101110111111, the process is 0011101110111111→00111011, 10111111→59, 191. Any 8-bit number can be represented as $bin_{7},bin_{6},bin_{5},bin_{4},bin_{3},bin_{2},b_{1},bin_{0}$ and can be converted into a decimal number D as $bin_{7}*2^{7}+bin_{6}*2^{6}+bin_{5}*2^{5}+bin_{4}*2^{4}+bin_{3}*2^{3}+bin_{2}*2^{2}+bin_{1}*2^{1}+bin_{0}*2^{0}$. The next step is to create a malicious code matrix. For this purpose, all malware substrings have been transformed into a one-dimensional vector of decimal numbers. Subsequently, a one-dimensional vector is transformed into a two-dimensional matrix of a certain width. The resultant two-dimensional matrix is then interpreted as a two-dimensional grayscale image. The graphical representation of the transformation process is depicted in the Figure 3.

**Figure 4:**
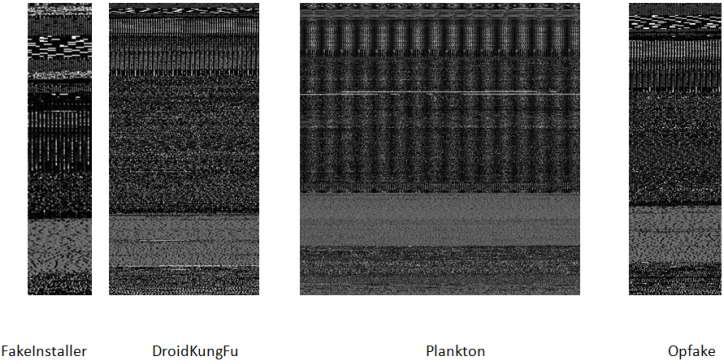
Illustration of malware images using the file sections of certificate (CR),Android manifest (AM), resource (RS), classes.dex (CL) of an APK.

Based on the empirical observations we have fixed the image widths according to the different image file size, as depicted in Table 1 [16]. It is to be noted that the height of malware image varies with the file size. Grayscale image visualization of Android families from DREBIN dataset is represented in Figure 4. The overall structure of grayscale images corresponds to various sections of an APK. Android malware images for twenty distinct families in the DREBIN dataset have been generated using fifteen different file structure combinations. These files are certificate (CR), Android manifest (AM), classes.dex (CL), and resource (RS).The combinations and associated samples of each class are illustrated in the Table 2. For example, the instances of malware images from various families with respect to CR+AM+RS+CL combinations are shown in Figure 4.

**Figure 5:**
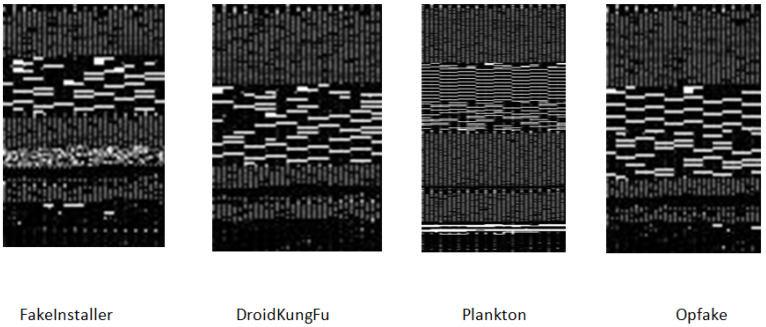
The fingerprint images of different malware family using file sections of Android manifest (AM) and resource (RS) of an APK structure.

The images of the malware generated from different malware families are visually comparable. They vary discernibly from images belonging to another family. For instance, in Figure 4 and Figure 5 variants of the FakeInstaller, DroidKungFu, Plankton, and Opfake malware family are shown. The images have different sizes and have visual dissimilarities. This is because they are created using the automation scripts or tools by the malware developers. Motivated by the visual similitude of malware images, we can classify and identify Android malware applications.

### 3.2. Experiment Design

#### 3.2.1. CNN Architectures

The proposed approach sees through binary information to discover and extract necessary insights for malware analysis. It paves the path for developing an effective malware classification system. CNN can attain high accuracy over challenging problems such as object detection, object classification and object recognition. They are a kind of special neural network for processing data that is known to have a grid-like topology. This could either be a one-dimensional time series data which is a grid of samples over time or two-dimensional image data. Every filter in CNN does some kind of operation to extract quality information from images. Filters in CNN play a very important role in extracting information from images. The detailed configuration of CNN architecture deployed during this experiment is briefed in Table 3. The description of each layer has been discussed below:

(a) **Convolutional Layer:** This is the first layer for CNN. At this layer, we convolve image or data using filters or kernels. Filters are small units that are to be applied through a sliding window. The depth of the filter is the same as that of input. For instance, a coloured image would have RGB values hence its depth would be set to three. In other words, a filter of depth 3 would be applied to it. The convolution operation involves taking the element-wise product of filters in the image and then summing those values for every sliding action. The output of the convolution of a 3D filter with a color image is a 2D matrix. It is important to note that convolution is not only applicable to images but can also convolve one-dimensional time-series data. In this experiment, the convolution layers are composed of 32, 128, and 256 with filters of size 7 × 7, 5 × 5, and 3 × 3 for the first, second, and third convolutional layer respectively.
(b) **Activation Function Layer:** An activation function is used to activate the neurons and send the signals further within the model. Weights and activation functions are important to transfer the signals through neurons. Rectified Linear Unit (ReLU) activation function prevents the vanishing gradient problem. It supports faster computation and less overhead as it does not compute exponentials and divisions. ReLU has been used to remove all the negative values from the output or matrix that we got through the convolution layer. It only activates a node if the input is above a certain threshold. While the input is below zero the output is also zero. When the input rises above the certain threshold it has a linear relationship with the dependent variable. The output of the ReLU activation function is fed to the pooling layer.
(c) **Pooling Layer:** It involves the downsampling of features to reduce the number of parameters during training. Typically, there are two hyper parameters introduced with the pooling layer. The first is the dimensions of the spatial extent. It is defined as the value of N for which we can take N × N feature representation and map to a single value. The second is the stride which is defined as how many features the sliding window should skip along the width and height of the malware image. In this experiment, the pooling layer uses a max filter of size 3 × 3, 3 × 3, and 2 × 2 for the first, second, and third convolutional layers respectively. It was moved across entire matrix resulted by ReLU layer. The maximum pixel value is taken from each window to shrink the malware image. All these layers were stacked up by adding more layers of convolution, ReLU, and pooling.
(d) **Batch Normalization Layer:** Batch normalization is used for stable learning of deep neural network. There is a significant problem in stable convergence in deep networks. This problem is caused by the vanishing and exploding gradient problems [64,65] and the different variants of activations within layers. The varying scale of different parameters cause bouncing in the gradient descent. In the forward propagation, it multiplicatively depends on each weight and activation function evaluation. The key point is that in the backward propagation, the partial derivative gets multiplied by the weights and the activation function derivatives. When the product of the weight and the activation function derivative is exactly one the gradients will either tend to increase or they will tend to decrease. This is partially caused by the fact that the activations in different layers have different variances. The distribution of input at each layer changes over training. Batch normalization is a way to address this issue by adding an additional batch normalization layer between the layers of the neural network. It ensures that the variances of the outputs of each layer are similar. Batch normalization normalizes not only the input features but also the features in each layer. This principle of normalization of the input features is carried through to all layers to ensure the most stable behaviour and faster convergence of the underlying algorithm.
(e) **Dropout Layer:** In the multilayer neural network, we often face an overfitting problem, also known as high variance problem. The Dropout layer in a neural network is used to solve the overfitting problem. Only a subset of features is selected from the input layer. Dropout randomly selects the neurons and deactivate them while learning the process. In a nutshell, deactivated neurons do not participate in the learning process. For every layer, a Dropout Ratio value is selected to be as 0.5.
(f) **Flatten Layer:** Flatten is a function or a library which converts the 2D image into 1D image. The flatten layer in the network takes the output from the previous layer and flattening it into a one-dimensional tensor. Basically, it takes the shrunk malware images and put it in a single list or vector.
(g) **Fully Connected/Dense Layer:** The output from the convolutional layers represents high-level features in data. Essentially the convolutional layers provide the meaningful low dimensional and somewhat invariant feature space whereas the fully connected layer learns a possible nonlinear function in that space. The output of a pooling layer has to be converted to a suitable input for the fully connected layers. The output of the pooling layer is a 3D feature map (a 3D volume of features). However, the input to a simple fully-connected feed-forward neural network is a one-dimensional feature vector. The features are usually very deep at this point because of the increased number of kernels that are introduced at every convolutional layer. Convolution, activation, and pooling layers can occur at many times before the fully connected layers and hence is the reason for the increased depth. To convert the 3D feature map into one dimension the output width and height has to be 1. This is done by flattening the 3D layer into a 1D vector. For classification problems, it involves introducing hidden layers and applying a softmax activation to the dense layers of neurons. In this paper, hidden dense layers D1, D2, and D3 have been added to the CNN architecture which has 50,100, and 200 neurons respectively. At the last, one more dense layer D4 is used as the output layer with 20 neurons. It classifies the malware images with respect to their families. Softmax is used as the activation function at the last layer.

#### 3.2.2. Machine Learning Algorithms

The machine learning algorithms such as KNN, SVM, and RF are applied to analyze the grayscale malware images using CNN features. The stated algorithms are discussed as follows:

(a) **KNN (K-NearestNeighbors):** KNN or K-Nearest Neighbor is a supervised classification algorithm. It identifies data points which are separated into several classes and predicts the class label for a new sample data point. It is a renowned method to classify data objects based on the closest training samples in a feature space. K in KNN refers to the number of nearest neighbors that the classifier will use to make its prediction. The unknown data points are classified by majority votes from chosen ‘K’ nearest neighbors. KNN uses the least distance measures such as Euclidean and Manhattan to find out the nearest neighbors. We have used Euclidean distance measure in this study.
(b) **SVM (Support Vector machine):**SVM is specific to supervised machine learning. The model based on supervised learning learns from the past input data and makes future predictions as output. SVM is primarily used for classification purposes, though it can also solve regression problem statements. In the SVM algorithm, support vectors are the extreme points in the dataset. The distance between the hyperplane and the support vectors should be as far as possible. Hyperplane has the maximum distance to the support vectors of any class. The distance between the support vectors of different classes is defined as a distance margin. Distance margin is calculated as the sum of D− and D+, where D− is the shortest distance from hyperplane to closest negative point and D+ is the shortest distance from hyperplane to the closest positive point. SVM aims to find the largest distance margin that leads to getting the optimal hyperplane. An optimal hyperplane produces good classification results. For the non-linear data or where hyperplane having a low or no margin, there is a high chance of misclassification of data points. In such scenarios, kernel functions are used to transform the data into a 2D or 3D array which makes it easy to split the data and classify. Kernel functions take the low dimensional feature space as input and transform into high dimensional feature space as output. Applications of the support vector machine are commonly used with it face detection, text and hypertext categorization, classification of images, and bioinformatics.
(c) **Random Forests:** The random forests algorithm is one of the most popular and powerful supervised machine learning algorithms that is capable of performing both regression and classification tasks. Random forests combine the simplicity of decision trees with flexibility resulting in a vast improvement in the accuracy. In general, the more trees in the forest, the more robust is the prediction. The use of multiple trees in random forests reduces the risk of overfitting. It runs efficiently and produces highly accurate predictions on large databases. Random forests can maintain accuracy even when there is a large proportion of data is missing. To classify a new object based on attributes each tree gives a classification result according to its defined rules. It can also be assumed that each tree cast its vote for classification. The random forests choose the classification class which has the most votes over all the other trees in the forests.

## 4. Results

Experiments were conducted on the DREBIN dataset. As the preprocessing step, the DREBIN dataset was transformed into malware images (discussed in previous sections). We have worked on the top 20 classes of the dataset, refer Table 2. The detailed algorithm of the proposed work is depicted in Algorithms 1–4. The machine with configuration Intel core i5 processor, 8G RAM, 2.7 Ghz clock speed and GPU was used for experimentation. Proposed SARVOTAM implementation includes the following steps. First, there is a need to train a deep convolutional neural network. It would actually be a coding network, and would extract the rich features from the malware images. These features represent high-level concepts for identification and classification of malware features. Finally, we design an efficient model to fuse the CNN features with machine learning algorithms. The results obtained are shown in Table 4. Support vector machine (SVM) is popular for classification, particularly for medical signal processing, image detection, face detection, geo and environmental sciences, and bioinformatics. For classification and recognition, great attention has been paid to the fusion of neural networks and SVM [66,67,68,69]. The benefits of their combination have been confirmed by many researchers for pedestrian detection [70], face recognition [71], and handwritten digit recognition [67].

**Figure 6:**
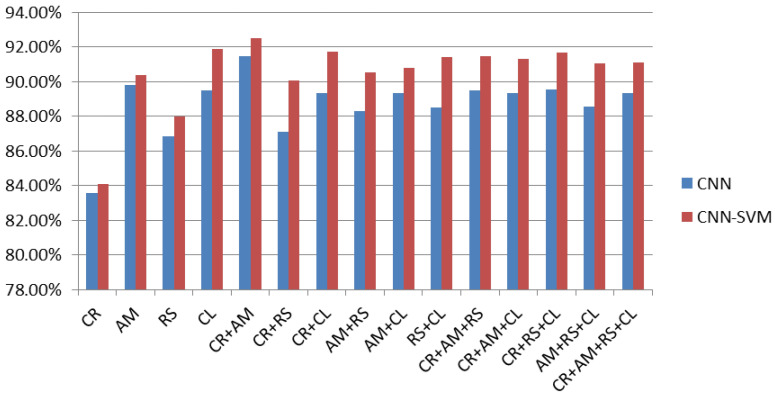
Fusion of CNN-SVM as substitute of CNN softmax layer.

**Figure 7:**
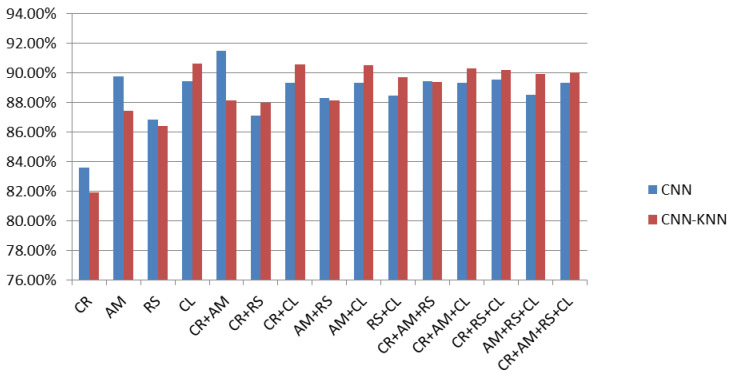
Fusion of CNN-KNN as substitute of CNN softmax layer.

**Figure 8:**
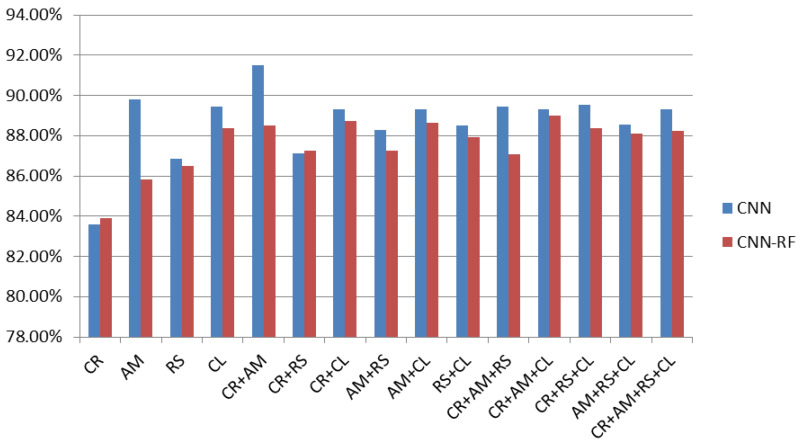
Fusion of CNN-RF as substitute of CNN softmax layer.

For classifier boosting, SVM, KNN, and RF are used as an alternative to softmax layer to enhance generalization ability of CNN. Stand-alone CNN architecture and other machine learning algorithms such as SVM, KNN, and RF were fused with CNN to augment the performance of proposed system on various combinations of malware images. As can be seen in Table 4, CR+AM were found to most precise features for identification and classification of Android malware. In case of generic CNN, an accuracy of 91.48% was recorded for classification of Android malware based on binary images. To further augment the classification accuracy of CNN its softmax layer was substituted with SVM, KNN, and RF. The results observed while substituting softmax layer with SVM, KNN, and RF are shown in Figure 6, Figure 7 and Figure 8 respectively.    
**Algorithm 1:** Classification of Android malware families    **Input**: Malicious aplications from DREBIN dataset    **Result**: Classification of Android malware families    **Step 1.** Import all the necessary libraries.    **Step 2.** An empty list is created for storing the training data.    train = [ ]    **Step 3.** Create list of 15 unique combinations.    combi_list=[‘CR’,‘AM’,‘RS’,‘CL’,‘CR+AM’,‘CR+RS’,‘CR+CL’,‘AM+RS’,‘AM+CL’,    ‘RS+CL’,‘CR+AM+RS’,‘CR+AM+CL’,‘CR+RS+CL’,‘AM+RS+CL’,‘CR+AM+RS+CL’]    **Step 4.** Load the pickle file from the local drive location in binary mode.    fw = **open**(‘local/content/drive/My Drive/allfiles.pckl’,‘rb’)    **Step 5.** Create the object of the file for further processing.    
obj=pickle.load(fw)
    **Step 6.** For every unique combination as stated in Step 3.    
[alldata,label,flist]=Fimg(obj,comb)
    
TRAINDATA=numpy.array(alldata)
    train_L=numpy.array(label)    model_cnn,train_all,test_label,pred_prob=cnn_model(TRAINDATA,train_L)    **Step 7.** Split the testing and training data and set up the features and labels.    [X_train, X_test, train_label, test_label] = train_test_split(train_all,    train_L, test_size=0.33, random_state=31,stratify=train_L)         feat_layer= K.function([model_cnn.layers[0].**input**],    [model_cnn.layers[12].output])    **for** i **in** **range**(0,**len**(X_train)):      feat=feat_layer([X_train[i:i+1,:,:,:]])[0]      **if**(i==0):        cnn_train=feat      **else**:        cnn_train=numpy.concatenate((cnn_train,feat),axis=0)    end **for**         **for** i **in** **range**(0,**len**(X_test)):      feat=feat_layer([X_test[i:i+1,:,:,:]])[0]      **if**(i==0):        cnn_test=feat      **else**:        cnn_test=numpy.concatenate((cnn_test,feat),axis=0)    end **for**    **Step 8.** Print the results.    Metrics=numpy.zeros((20,4))    CONFUSION=[]    AUC=[]    **print**(’CNN results’)    Metrics[0:4,:],conf,auc_value=eval_classi(cnn_train,cnn_test,    train_label,test_label,‘ORG_CNN_’)    CONFUSION.append(conf)    AUC.append(auc_value)**Algorithm 2:** Loading the malware families from the path using the above procedure    **Input**: Call made from Algorithm 1 to read the families    **Result**: Classes with count, names and labels of the family         Procedure read_family():                tl=pd.read_csv(‘local/content/drive/My Drive/sha256_family.csv’,           header=None)           tl.drop(tl.index[[0,0]], inplace = True)           labelencoder_X = LabelEncoder()           Nlabels=labelencoder_X.fit_transform(tl[1])           qa=pd.value_counts(Nlabels)           Sclasses=(qa[0:20].index).values           allnames=tl[0].tolist()           **return** Sclasses,allnames,Nlabels         end procedure         procedure get_label(fname,allnames,Nlabels,Sclasses):              idxC=allnames.index(fname)         idxclass=Nlabels[idxC]         TMP=numpy.where(Sclasses==idxclass)         **return** **int**(TMP[0])         end procedure

**Algorithm 3:** Training of CNN model    **Input**: Malware images    **Result**: Trained CNN model    **Step 1.** To train the CNN model important libraries such as Conv2D and MaxPooling2D,    Activation, Dropout, Flatten, and Dense are imported.    **Step 2.** Object of sequential function is created which defines the model name of the neural    network.    model = tf.keras.Sequential()    **Step 3.** The next step is to build and train the CNN on the malware images of different families.     
Since we are dealing with the 2D malware grayscale images, we added the first convolutional    
layer to the model which is represented as the Conv2D layer.    model.add(Conv2D(32,(7,7),strides=1,padding=‘valid’, kernel_initializer=    ‘glorot_uniform’, input_shape=(108,108,1),use_bias=True))    **Step 4.** In the convolution layer, each feature will move throughout the entire image and the    pixel value of the image gets multiplied with that of the corresponding pixel value of the filter    
adding them up and dividing by the total number of pixels to get the output.    **Step 5.** ReLU activation function is applied as we want to remove all the negative values from    the output or matrix that we got through the convolution layer.    tf.keras.layers.ReLU(max_value=None, negative_slope=0.0, threshold=0.0)    
**Step 6.** The output of the ReLU activation function is fed to the MaxPooling layer. The pooling    
layer uses a max filter of size 3 × 3, 3 × 3, and 2 × 2 for the first, second, and third    
convolutional layers respectively.    model.add(MaxPooling2D(pool_size=(3,3)))    
**Step 7.** Batch normalization is applied for the stable learning of the network    model.add(BatchNormalization())    
**Step 8.** The Dropout layer in a neural network is used to solve the overfitting problem. The    value is selected to be as 0.5.    model.add(Dropout(0.5))    
**Step 9.** More layers of convolution, ReLU, pooling, batch normalization, and dropout are    stacked up.    model.add(Conv2D(128,(5,5),strides=1,padding=‘valid’,    kernel_initializer=‘glorot_uniform’,use_bias=True))    tf.keras.layers.ReLU(max_value=None, negative_slope=0.0, threshold=0.0)    model.add(MaxPooling2D(pool_size=(3,3)))    model.add(BatchNormalization())    model.add(Dropout(0.5))         model.add(Conv2D(256,(3,3),strides=1,padding=‘valid’,    kernel_initializer=‘glorot_uniform’,use_bias=True))    tf.keras.layers.ReLU(max_value=None, negative_slope=0.0, threshold=0.0)    model.add(MaxPooling2D(pool_size=(2,2)))    model.add(BatchNormalization())    model.add(Dropout(0.5))    
**Step 10.** The flatten layer is used in the network that takes the output from the previous layers    and flattening it into a one-dimensional tensor. Shrunk malware images are put it in a single    list or vector.    model.add(Flatten())    **Step 11.** Further, malware images fed into a fully connected layer/dense layer. Three dense    layers D1, D2, and D3 have been added to the CNN architecture which has 50,100, and 200    neurons respectively.    model.add(Dense(50,kernel_initializer=‘glorot_uniform’,use_bias=True))    tf.keras.layers.ReLU(max_value=None, negative_slope=0.0, threshold=0.0)    model.add(Dense(100,kernel_initializer=‘glorot_uniform’,use_bias=True))    tf.keras.layers.ReLU(max_value=None, negative_slope=0.0, threshold=0.0)    model.add(Dense(200,kernel_initializer=‘glorot_uniform’,use_bias=True))    tf.keras.layers.ReLU(max_value=None, negative_slope=0.0, threshold=0.0)         
**Step 12.** Apply one more dense layer as the output layer with 20 nodes. It classifies the    malware images with respect to their families.    
**Step 13.** Apply the activation function of softmax in the last layer.    model.add(Dense(20,activation=‘softmax’))    
**Step 14.** In the compilation phase of the model, apply adam optimizer and loss as categorical    cross-entropy.    model.**compile**(loss=‘sparse_categorical_crossentropy’, optimizer=’adam’,    metrics=[‘accuracy’])    
**Step 15.** The array is specified with the single string accuracy as the metrics. To train the     model, function model.fit() generator is called. Model is trained for 100 epochs.    model.fit(datagen1.flow(X_train, train_label, batch_size=16),    steps_per_epoch=**len**(X_train)//16,epochs=100,verbose=1)
 


**Algorithm 4:** Transformation of malware binary into images    
**Input**: Malware binary    
**Result**: Malware images of the family depending upon the combination    procedure transform(Family[i]), comb):                      **if**(comb==1)                 Result = make_image(Famliy[i], extract certificate files                 **from** each sample of Family[i])                      **elif**(comb==2)                 Result = make_image(Famliy[i], extract android manifest                 files **from** each sample of Family[i])                      **elif**(comb==3)                 Result = make_image(Famliy[i], extract resource files                 **from** each sample of Family[i])                      **elif**(comb==4)                 Result = make_image(Famliy[i], extract classes.dex files                 **from** each sample of Family[i])                      **elif**(comb==5)                 Result = make_image(Famliy[i], extract certificate **and**                   android manifest files **from** each sample of Family[i])                      **elif**(comb==6)                 Result = make_image(Famliy[i], extract certificate **and**                   resource files **from** each sample of Family[i])                      **elif**(comb==7)                 Result = make_image(Famliy[i], extract certificate **and**                   classes.dex files **from** each sample of Family[i])                      **elif**(comb==8)                 Result = make_image(Famliy[i], extract android manifest                 **and** resource files **from** each sample of Family[i])                      **elif**(comb==9)                 Result = make_image(Famliy[i], extract android manifest                 **and** classes.dex files **from** each sample of Family[i])                      **elif**(comb==10)                 Result = make_image(Famliy[i], extract resource **and**                   classes.dex files **from** each sample of Family[i])                      **elif**(comb==11)                 Result = make_image(Famliy[i], extract certificate,                 android manifest, **and** resource files **from** each sample                 of Family[i])                      **elif**(comb==12)                 Result = make_image(Famliy[i], extract certificate,                 android manifest, **and** classes.dex files **from** each sample                 of Family[i])                      **elif**(comb==13)                 Result = make_image(Famliy[i], extract certificate,                 resource, **and** classes.dex files **from** each sample                 of Family[i])                      **elif**(comb==14)                 Result = make_image(Famliy[i], extract android manifest,                   resource, **and** classes.dex **from** each sample of Family[i])                      **elif**(comb==15)                 Result = make_image(Famliy[i], extract certificate,                 android manifest, resource, **and** classes.dex **from** each                 sample of Family[i])         End procedure         procedure make_image(ar,filesizelist,widthlist):             ar_len=**len**(ar)/1024        width=0        **for** cidx **in** **range**(1,**len**(filesizelist)):            **if**(ar_len>=filesizelist[cidx-1] **and** ar_len<filesizelist[cidx]):                width=widthlist[cidx-1]        **if**(width==0):            width=1024        rem1=**len**(ar)\%width        n=array.array("B")        n.frombytes(ar)        a=array.array("B")        a=n[0:**len**(ar)-rem1]        **if** (**len**(a)<width):            **return** numpy.array([])        img=numpy.reshape(a,(**int**(**len**(a)/width),width))        img=numpy.uint8(img)        **return** img     
End procedure


It was observed that fusion of CNN-SVM outperformed rest of the softmax layer substitutes. An improvement of classification accuracy has been observed for entire fifteen combinations of malware image sections. For thirteen combinations, CNN-SVM is able to achieve accuracy in the window 90% to 93%, as shown in Figure 6. The highest accuracy of 92.59% is observed using CR+AM combination of malware images. The increase in accuracy ranges from 0.50% to 3%.

Using KNN within CNN as softmax layer resulted in marginal increase in CNN accuracy that too in case of a few image sections. A decrease in accuracy was also observed with respect to the combination of CR and AM. The average classification results of CNN and CNN-KNN is observed between 88.66% and 88.76% respectively. Detailed performance of CNN-KNN fusion is depicted in Figure 7.

Integrating RF with CNN resulted in poorest performance in comparison to SVM and KNN. CNN-RF, performed poorly as shown in Figure 8.

Table 5 shows the comparison of proposed work with that of state-of-the-art proposals. The detailed runtime performance metrics such as memory-consumption, total execution time and time spent to identify a possible APK as malware using different combinations of malware images is shown in Table 6.

**Figure 9:**
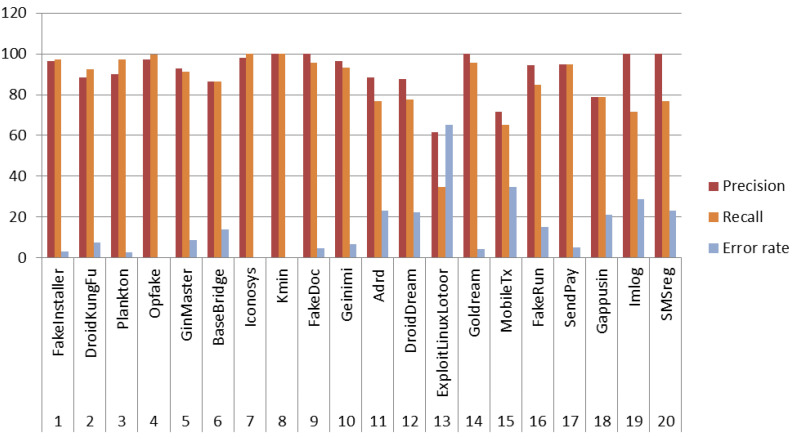
Precision, Recall, and Error rate of CNN-SVM in top 20 malware families of Drebin Dataset.

**Figure 10:**
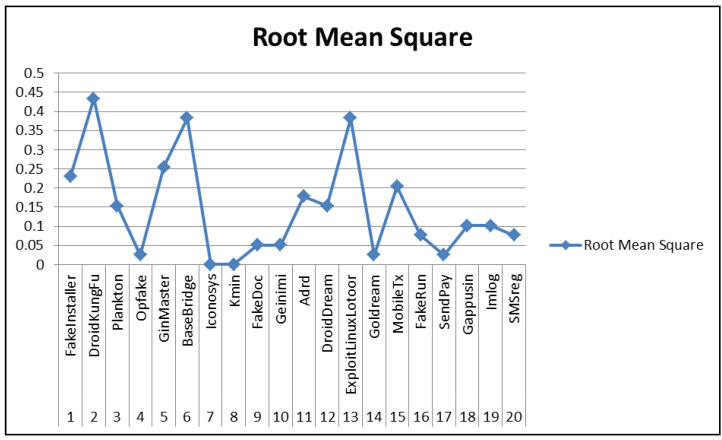
Root mean square analysis of top 20 malware families of Drebin Dataset.

In our work, CNN-SVM performed well on comparison to generic CNN architecture and other substitutes of softmax layer for 100 epochs. The detailed confusion matrix and other performance metrics are presented in Table 7 and Figure 9 respectively. Among all classifier combination, CNN infusion with SVM perfomed well and particularly showed high precision and recall for the Android malware families Kmin, GoldDream, FakeDoc, Iconosys, Opfake, and FakeInstaller. CNN-SVM enhanced the performance in malware classification and attained the accuracy of 92.59% using CR+AM images, as discussed earlier. The performance of CNN-SVM showed low precision and recall for the malware families such as ExploitLinuxLootor, MobileTx, Gappusin, and BaseBridge. This is mainly due to the reason that these Android malware families contain less number of samples as compared to other families. Malware family SendPay attained equal precision and recall of 0.94. The error rate of malware families such as Kmin and Iconosys is 0. It means that the model learned the actual behavior of these malware families. The highest error rate was observed for the malware families such as ExploitLinuxLootor, MobileTx, Imlog, SMSreg, DroidDream, and Gappusin. The probable reason for low performance of the proposed method in case of malware like ExploitLinuxLotoor was the small number of samples within the training dataset. Such malware families are meant to exploit a rooted Android device the most (where admin rights of the device are with used and not with stock Android provider or proprietor). It alters its signature after attaining root access of the device, till it does so, the malware file tries to look legitimate to the extent possible. Evaluating the proposed method on rooted and non-rooted devices opens a new horizon for this research. It is to be noted that samples of malware families Imlog and SMSreg get highly misclassified to other families but achieved the precision as high as 100%. This depicts that images of these families are highly different from other malware families. The classification achieved low error rate for malware families Opfake, Plankton, FakeInstaller, Golddream, Fakedream, SendPay, and Geinimi which ranged from 2% to 6%. The root mean square analysis was done to measure the error rate of the proposed method. It was calculated for every malware family as shown in Figure 10. The value is found to be in between 0 to 0.45. A comparison of the proposed model with that of Visual Geometry Group (VGG16) typic nertwork was done. VGG16 is a typic convolutional neural network which is adopted from the VGG family. VGG16 network architecture has been previously used to solve multi-class malware familial classification problem [78,79]. A comparison of classification accuracy of SARVOTAM and VGG16 on different malware image combinations is presented in Table 8. As per the recorded observations, proposed CNN structure(s) attained better accuracy than VGG16. The average accuracy of VGG16 is visibaly less than the average accuracy of SARVOTAM. VGG16 attained an average accuracy of 86.02% whereas, for CNN-SVM, CNN, CNN-KNN, and CNN-RF it was recorded at 89.96%, 88.66%, 87.50%, and 86.78% respectively. The classification execution time and RAM usage based on different malware images combination using the VGG16 network and SARVOTAM is also depicted in Table 9.

The information in the Table 9 reveals that VGG16 is heavy on time and memory. The average classification time for all malware image combinations is recorded to be as 1720.72 s. The SARVOTAM model attained the average classification time as low as 972.78 s. The average RAM usage is observed to be 59.67% for VGG16 whereas, for SARVOTAM, it is recorded as 53.09%. The performance of SARVOTAM was best recorded for the malware image combination CR+AM. It utilized 37.33% of the total RAM available and took 840.22 s to classify Android malware applications. The malware image combination CR+AM attained a classification accuracy of 92.59% using CNN-SVM. The malware images generated using only CR and AM files took less time and RAM than CR+AM but their highest accuracy was recorded as 83.58% using CNN and 90.18% using CNN-SVM respectively which was lesser than CR+AM. CR+AM proved to be the lightweight combination to classify applications. VGG16 also attained a high accuracy of 90.57% on CR+AM malware images but at the same time consumes more memory and time. It almost took double the time and 4.33% more consumption of memory as taken by CNN-SVM.

## 5. Conclusions and Future Scope

This manuscript concludes the fact that certificate and Android manifest (CR+AM) are most suited features for malware identification and classification. Generic CNN attained a maximum accuracy of 91.48%. The softmax layer of CNN was augmented for classification purposes using SVM, KNN and RF. The combination of CNN and SVM was found to be most suited and even surpassed generic CNN in identification and classification of Android malware families. CNN-SVM achieved the classification acuracy of 92.59%. Following common sense, one may try to identify and classify malware using entire of the features for malware images. This may demand additional hardware resources, time and complex comparisons for identification of malware features. On the other hand, CR+AM offer a light weight and much precise option for malware identification. The proposed methodology is primarily focused on identification and classification of malware images using feature extraction techniques instead of static and dynamic analysis of malware applications. Malware authors employ automation tools to generate dynamic payloads and inject them into the applications. It was noticed that the malware families hard coded with dynamic payloads or some obfuscated code, tend to generate similar malware images. Therefore, a visual similarity between malware images from the same malware family is anticipated. The scope of this experiment was limited to evaluate the performance of the proposed model using malware images. Obfuscation images may look legitimate but they differ with respect to the access rights, resource utilization and other attributes related to APKs, this is why they do not look completely similar to legitimate Android applications and can be classified using proposed method. We will look forward to attune the proposed methodology to be used alongside static and dynamic analysis as future scope of this research. We also intend to investigate the effect of data augmentation and feature fusion strategy. Also, the transformation of malware images into color images and fine-tuning of pre-trained typic CNNs need to be further explored for the classification of Android malware images.

## Figures and Tables

**Table 1:** Fixed Image Width According to File Size.

File Size	Width
<50 KB	64
50 KB~100 KB	128
100 KB~200 KB	256
200 KB~500 KB	512
500 KB~1000 KB	1024

**Table 2:** The combinations and associated instances of each malware class from DREBIN dataset.

Name	Classes	CR *	AM *	RS *	CL *	CR+AM	CR+RS	CR+CL	AM+RS	AM+CL	RS+CL	CR+AM+RS	CR+AM+CL	CR+RS+CL	AM+RS+CL	CR+AM+RS+CL
**FakeInstaller **	1	360	925	925	925	925	925	925	925	925	925	925	925	925	925	925
**DroidKungFu**	2	236	666	666	666	666	666	666	666	666	666	666	666	666	666	666
**Plankton**	3	439	625	625	625	625	625	625	625	625	625	625	625	625	625	625
**Opfake**	4	5	613	613	613	613	613	613	613	613	613	613	613	613	613	613
**GinMaster**	5	30	339	339	339	339	339	339	339	339	339	339	339	339	339	339
**BaseBridge**	6	13	329	329	329	329	329	329	329	329	329	329	329	329	329	329
**Iconosys**	7	152	152	152	152	152	152	152	152	152	152	152	152	152	152	152
**Kmin**	8	4	147	147	147	147	147	147	147	147	147	147	147	147	147	147
**FakeDoc**	9	107	132	132	132	132	132	132	132	132	132	132	132	132	132	132
**Geinimi**	10	91	91	91	91	91	91	91	91	91	91	91	91	91	91	91
**Adrd**	11	88	91	91	91	91	91	91	91	91	91	91	91	91	91	91
**DroidDream**	12	63	81	81	81	81	81	81	81	81	81	81	81	81	81	81
**ExploitLinuxLotoor**	13	39	69	69	69	69	69	69	69	69	69	69	69	69	69	69
**MobileTx**	14	20	69	69	69	69	69	69	69	69	69	69	69	69	69	69
**Glodream**	15	59	69	69	69	69	69	69	69	69	69	69	69	69	69	69
**FakeRun**	16	27	61	61	61	61	61	61	61	61	61	61	61	61	61	61
**SendPay**	17	22	59	59	59	59	59	59	59	59	59	59	59	59	59	59
**Gappusin**	18	51	58	58	58	58	58	58	58	58	58	58	58	58	58	58
**Imlog**	19	6	43	43	43	43	43	43	43	43	43	43	43	43	43	43
**SMSreg**	20	14	40	40	41	40	40	41	40	41	41	40	41	41	41	41
	**All instances**	1826	4659	4659	4660	4659	4659	4660	4659	4660	4660	4659	4660	4660	4660	4660

* CR->Certificate, AM->AndroidManifest, RS->Resource, CL->Classes.dex.

**Table 3:** Detailed Configuration of CNN Architecture.

Layer Number	Layer Type	Hyperparameters	
Layer 1	Convolution Layer	Filter Size	7 × 7
		Number of Filters	32
		Activation Layer	Relu
Layer 2	Pooling Layer	Pool Size	3 × 3
		Pooling type	Max-Pooling
Layer 3	Batch Normalization Layer		
Layer 4	Dropout Layer	Rate	0.5
Layer 5	Convolution Layer	Filter Size	5 × 5
		Number of Filters	128
		Activation Layer	Relu
Layer 6	Pooling Layer	Pool Size	3 × 3
		Pooling type	Max-Pooling
Layer 7	Batch Normalization Layer		
Layer 8	Dropout Layer	Rate	0.5
Layer 9	Convolution Layer	Filter Size	3 × 3
		Number of Filters	256
		Activation Layer	Relu
Layer 10	Pooling Layer	Pool Size	2 × 2
		Pooling type	Max-Pooling
Layer 11	Batch Normalization Layer		
Layer 12	Dropout Layer	Rate	0.5
Layer 13	Flatten Layer		
Layer 14	Dense Layer (D1)	Activation Layer	Relu
		Neurons	50
Layer 15	Dense Layer (D2)	Activation Layer	Relu
		Neurons	100
Layer 16	Dense Layer (D3)	Activation Layer	Relu
		Neurons	200
Layer 17	Dense Layer (D4)	Activation Layer	Softmax
		Neurons	20

**Table 4:** Accuracy of generic and augmented CNN on 15 different combinations of grayscale malware images.

	Image Combination	CNN	CNN-SVM	CNN-KNN	CNN-RF
1	**CR**	83.58%	82.92%	77.11%	83.42%
2	**AM**	89.79%	90.18%	83.94%	84.85%
3	**RS**	86.86%	88.56%	86.02%	84.53%
4	**CL**	89.46%	90.57%	89.40%	87.58%
5	**CR+AM**	91.48%	92.59%	86.93%	87.52%
6	**CR+RS**	87.12%	89.47%	86.80%	85.89%
7	**CR+CL**	89.33%	90.25%	89.01%	88.43%
8	**AM+RS**	88.29%	89.47%	87.78%	84.98%
9	**AM+CL**	89.33%	90.83%	89.79%	88.69%
10	**RS+CL**	88.49%	90.96%	89.34%	87.58%
11	**CR+AM+RS**	89.46%	90.77%	88.75%	85.50%
12	**CR+AM+CL**	89.33%	90.51%	88.49%	88.82%
13	**CR+RS+CL**	89.53%	90.90%	89.66%	88.17%
14	**AM+RS+CL**	88.55%	90.70%	89.86%	87.97%
15	**CR+AM+RS+CL**	89.33%	90.70%	89.60%	87.84%

**Table 5:** Comparative summary of SARVOTAM with previous studies.

Study	Classification of Android Malware Families	Automatic Extraction of Features through Deep Learning	Features	Model	Time (s)	Environment
[42]	Yes	No	Control flow graph	Single linkage clustering	Not specified	Not specified
[44]	Yes	No	Call graph, Application programming interface (API)	Naive Bayes (NB), Support Vector Machine (SVM), Decision Tree (DT), Random Forest (RF)	19.8	Corei5, 6 G RAM
[72]	No	No	Permissions, events generated by monkey tool	Recurrent Neural Network (RNN), Long Short Term Memory (LSTM)	Not specified	Not specified
[73]	Yes	No	Information flow between APIs	Semantic-based approach	175.88	Xeon, 128 G RAM
[74]	No	No	System calls	Convolutional Neural Network (CNN)	Executed app for 60 s	Not specified
[75]	Yes	No	Application programming interface	Visualization and similarity-based	Not specified	Not specified
[6]	Yes	No	Permissions, Package names, Intents, Information flow between APIs	C4.5	95.2	8-core, 64 G RAM
[76]	Yes	No	Permissions, API	Deep belief network	Not specified	Not specified
[77]	No	No	System calls into feature vectors	K-means	Not stated	Not specified
[43]	Yes	No	Network, System calls, File system access, Binder transactions	SVM	Not specified	Not specified
Our method (SARVOTAM)	Yes	Yes	CNN features extacted from Lightweight malware images	CNN, CNN-SVM, CNN-KNN, CNN-RF	0.55	Core i5, 8 G RAM

**Table 6:** A comparison of runtime metrics to showcase resource usage and execution time taken.

S.No.	Combination	RAM Usage (in %)	Execution Time (s)	Average Time per App (s)
1	**CR**	36.50	228.89	0.15
2	**AM**	37.33	751.33	0.49
3	**RS**	48.42	880.98	0.57
4	**CL**	49.33	1069.71	0.70
5	**CR+AM**	44.42	840.22	0.55
6	**CR+RS**	49.75	953.16	0.62
7	**CR+CL**	51.17	1074.94	0.70
8	**AM+RS**	57.92	855.42	0.56
9	**AM+CL**	56.58	1052.11	0.68
10	**RS+CL**	57.75	1060.74	0.69
11	**CR+AM+RS**	57.25	896.58	0.58
12	**CR+AM+CL**	63.58	1088.45	0.71
13	**CR+RS+CL**	54.33	1207.43	0.79
14	**AM+RS+CL**	63.67	1153.69	0.75
15	**CR+AM+RS+CL**	68.42	1478.16	0.96

**Table 7:** A confusion matrix of top 20 malware families.

S.No.	FamilyName	FakeInstaller	DroidKungFu	Plankton	Opfake	GinMaster	BaseBridge	Iconosys	Kmin	FakeDoc	Geinimi	Adrd	DroidDream	ExploitLinuxLotoor	Glodream	MobileTx	FakeRun	SendPay	Gappusin	Imlog	SMSreg
**1 **	**FakeInstaller**	296	1	0	3	0	3	1	0	0	0	0	0	0	0	0	0	0	1	0	0
**2**	**DroidKungFu**	0	203	5	2	1	4	0	0	0	0	1	0	1	0	2	0	0	1	0	0
**3**	**Plankton**	0	3	200	0	1	0	0	0	0	0	0	1	0	0	0	1	0	0	0	0
**4**	**Opfake**	1	0	0	201	0	0	0	0	0	0	0	0	0	0	0	0	0	0	0	0
**5**	**GinMaster**	0	3	3	0	102	1	0	0	0	0	0	0	2	0	0	0	1	0	0	0
**6**	**BaseBridge**	3	6	4	0	0	94	0	0	0	1	0	0	1	0	0	0	0	0	0	0
**7**	**Iconosys**	0	0	0	0	0	0	50	0	0	0	0	0	0	0	0	0	0	0	0	0
**8**	**Kmin**	0	0	0	0	0	0	0	49	0	0	0	0	0	0	0	0	0	0	0	0
**9**	**FakeDoc**	0	0	1	0	0	0	0	0	42	0	0	0	0	0	1	0	0	0	0	0
**10**	**Geinimi**	0	0	2	0	0	0	0	0	0	28	0	0	0	0	0	0	0	0	0	0
**11**	**Adrd**	0	3	1	0	0	0	0	0	0	0	23	0	0	0	3	0	0	0	0	0
**12**	**DroidDream**	1	0	0	1	1	2	0	0	0	0	0	21	0	0	0	0	0	1	0	0
**13**	**ExploitLinuxLotoor**	3	5	1	0	2	3	0	0	0	0	0	1	8	0	0	0	0	0	0	0
**14**	**Goldream**	0	0	0	0	0	1	0	0	0	0	0	0	0	22	0	0	0	0	0	0
**15**	**MobileTx**	1	3	1	0	1	1	0	0	0	0	1	0	0	0	15	0	0	0	0	0
**16**	**FakeRun**	0	0	2	0	0	0	0	0	0	0	0	1	0	0	0	17	0	0	0	0
**17**	**SendPay**	0	1	0	0	0	0	0	0	0	0	0	0	0	0	0	0	18	0	0	0
**18**	**Gappusin**	0	1	1	0	0	0	0	0	0	0	1	0	1	0	0	0	0	15	0	0
**19**	**Imlog**	0	1	1	0	1	0	0	0	0	0	0	0	0	0	0	0	0	1	10	0
**20**	**SMSreg**	2	0	0	0	1	0	0	0	0	0	0	0	0	0	0	0	0	0	0	10

**Table 8:** Observed accuracy of SARVOTAM in comparison to VGG16 typic network.

S.No.	Image Combination	SARVOTAM				Typic Network
		CNN	CNN-SVM	CNN-KNN	CNN-RF	VGG16
1	**CR **	83.58%	82.92%	77.11%	83.42%	78.27%
2	**AM**	89.79%	90.18%	83.94%	84.85%	85.76%
3	**RS**	86.86%	88.56%	86.02%	84.53%	82.12%
4	**CL**	89.46%	90.57%	89.40%	87.58%	87.23%
5	**CR+AM**	91.48%	92.59%	86.93%	87.52%	90.57%
6	**CR+RS**	87.12%	89.47%	86.80%	85.89%	88.91%
7	**CR+CL**	89.33%	90.25%	89.01%	88.43%	89.34%
8	**AM+RS**	88.29%	89.47%	87.78%	84.98%	86.78%
9	**AM+CL**	89.33%	90.83%	89.79%	88.69%	84.43%
10	**RS+CL**	88.49%	90.96%	89.34%	87.58%	84.37%
11	**CR+AM+RS**	89.46%	90.77%	88.75%	85.50%	87.67%
12	**CR+AM+CL**	89.33%	90.51%	88.49%	88.82%	86.81%
13	**CR+RS+CL**	89.53%	90.90%	89.66%	88.17%	84.56%
14	**AM+RS+CL**	88.55%	90.70%	89.86%	87.97%	89.29%
15	**CR+AM+RS+CL**	89.33%	90.70%	89.60%	87.84%	84.32%

**Table 9:** Recorded RAM utilization and execution time for SARVOTAM and VGG16 while classifying Android malware families.

S.No.	Combination	VGG16 Run Time Performance		SARVOTAM Run Time Performance	
		RAM Usage (in %)	Execution Time (in secs)	RAM Usage (in %)	Execution Time (in secs)
1	**CR**	44.50	1935.51	36.50	228.89
2	**AM**	48.67	1722.41	37.33	751.33
3	**RS**	70.33	1548.42	48.42	880.98
4	**CL**	55.08	1635.32	49.33	1069.71
5	**CR+AM**	48.75	1754.86	44.42	840.22
6	**CR+RS**	49.58	1724.42	49.75	953.16
7	**CR+CL**	56.5	1680.31	51.17	1074.94
8	**AM+RS**	55.42	1535.78	57.92	855.42
9	**AM+CL**	55.17	1418.45	56.58	1052.11
10	**RS+CL**	62.92	1656.43	57.75	1060.74
11	**CR+AM+RS**	63.5	1771.69	57.25	896.58
12	**CR+AM+CL**	63.75	1619.16	63.58	1088.45
13	**CR+RS+CL**	73.25	1834.11	54.33	1207.43
14	**AM+RS+CL**	73.42	1795.91	63.67	1153.69
15	**CR+AM+RS+CL**	74.33	2178.12	68.42	1478.16
